# Studies Related to the Involvement of EsA in Improving Intestinal Inflammation in Acute Pancreatitis via the NF-*κ*B Pathway

**DOI:** 10.1155/2024/9078794

**Published:** 2024-04-01

**Authors:** CuiPing Pan, ChunXiang Zhang, YiJie Li, Jie Cao, ShiWei Liang, HaiCheng Fang, Ying Liu

**Affiliations:** ^1^Department of Gastroenterology, The Second Affiliated Hospital of Guilin Medical University, Guilin 541100, China; ^2^Guangxi Health Commission Key Laboratory of Glucose and Lipid Metabolism Disorders, The Second Affiliated Hospital of Guilin Medical University, Guilin 541100, China

## Abstract

**Background:**

Acute pancreatitis (AP) is a clinically frequent acute abdominal condition, which refers to an inflammatory response syndrome of edema, bleeding, and even necrosis caused by abnormal activation of the pancreas's own digestive enzymes. Intestinal damage can occur early in the course of AP and is manifested by impaired intestinal mucosal barrier function, and inflammatory reactions of the intestinal mucosa, among other factors. It can cause translocation of intestinal bacteria and endotoxins, further aggravating the condition of AP. Therefore, actively protecting the intestinal mucosal barrier, controlling the progression of intestinal inflammation, and improving intestinal dynamics in the early stages of AP play an important role in enhancing the prognosis of AP.

**Methods:**

The viability and apoptosis of RAW264.7 cells treated with Esculentoside A (EsA) and/or lipopolysaccharide were detected using 3-(4,5-dimethylthiazol-2-yl)-2,5-diphenyltetrazolium bromide (MTT) and flow cytometry, respectively. The expression of apoptosis-related proteins and NF-*κ*B signaling pathway-related proteins were detected by western blot (WB). An enzyme-linked immunosorbent assay was used to measure TNF-*α* and IL-6 secretion.

**Results:**

In vitro experiments demonstrated that EsA not only promoted the apoptosis of inflammatory cells but also reduced the secretion of TNF-*α* and IL-6 in a dose-dependent manner. Additionally, it inhibited the activation of the NF-*κ*B signaling pathway by decreasing the expression of phosphorylated-p65(p-p65) and elevating the expression of I*κ*B*α*. Similarly, in vivo experiments using a rat AP model showed that EsA inhibited the expression of p-p65 elevating the expression of I*κ*B*α* in the intestinal tissues of the rat AP model and promoting the apoptosis of inflammatory cells in the intestinal mucosa in vivo experiments, while improving the pathological outcome of the pancreatic and intestinal tissues.

**Conclusion:**

Our results suggest that EsA can reduce intestinal inflammation in the rat AP model and that EsA may be a candidate for treating intestinal inflammation in AP and further arresting AP progression.

## 1. Introduction

Acute pancreatitis (AP) is a common clinical condition characterized by acute abdominal pain [1, 2]. Various factors stimulate the pancreas to secrete a large number of digestive enzymes, leading to self-digestion, inflammation, edema, bleeding, and even necrosis of the pancreas or surrounding adipose tissue. In the course of AP, various inflammatory factors trigger a cascade reaction of inflammatory mediators through a “trigger-like effect”, leading to systemic inflammatory response syndrome (SIRS) and multiple organ dysfunction syndrome (MODS) [3]. The intestine and lungs are considered the most common target organs for organ function damage during AP. Intestinal and pulmonary function damage is the primary cause of early death in AP, with a mortality rate as high as 60% [4].

Intestinal damage can occur in the early course of AP and manifests itself as impaired intestinal mucosal barrier function, which in turn causes an inflammatory response in the intestinal mucosa [5, 6]. Intestinal inflammation is connected to the pancreas and then passes through the intestinal inflammation into the lungs via the body circulation and mesenteric lymphatic pathway, ultimately leading to and exacerbating acute lung inflammation. Cascade release of inflammatory mediators and cytokines, which can lead to SIRS and MODS [7–11], and lead to distant infection and abdominal compartment syndrome, which can aggravate the condition of AP. Therefore, it is important to protect intestinal mucosal barrier, control the progress of intestinal inflammation, and improve intestinal motility at the early stage of AP to improve the prognosis of AP.

So far, there are few treatments related to intestinal inflammation in AP, and most of them inhibit the secretion of pancreatic enzymes through hormones and somatostatin to reduce pancreatic self-digestion. However, there is no direct treatment effect for intestinal damage. In the early stage of AP, intestinal inflammation further spreads to other organs to cause damage and eventually develops into MODS. Therefore, it is very important to treat intestinal inflammation in AP directly in the early stage to prevent the further spread of AP.

Esculentoside A (EsA) is a saponin extracted from the root of *Phytolacca acinosa*, a natural product, which has a wide range of pharmacological effects, such as anti-inflammatory, diuretic, and immune regulation. The latest research found that EsA can regulate the NF-*κ*B and MAPK signal pathway as well as the Nrf-2 signal pathway to inhibit inflammation [12]. Ma et al. [13] found that EsA can treat nephritis through anti-inflammatory and apoptosis-promoting mechanisms. Although EsA plays an anti-inflammatory role in many diseases, its role in intestinal inflammation of AP is still unknown. Therefore, the purpose of this study was to explore the effect of EsA on lipopolysaccharide (LPS)-induced RAW264.7 inflammatory cells and the role and mechanism of EsA in AP in rats.

In this study, we used LPS to treat RAW264.7 cells to establish inflammatory cells models, and used cerulein and LPS to construct AP rat models. We studied the effect of EsA on apoptosis and inflammation both in vivo and in vitro, in order to confirm that EsA can be used as an effective drug to treat intestinal inflammation in AP. To the best of our knowledge, this is the first study on the possible beneficial effects of EsA on intestinal inflammation in AP. Our research findings may provide a theoretical basis for further understanding the inhibitory effect of EsA on intestinal inflammation in AP.

## 2. Materials and Methods

### 2.1. Animals

SD rats were purchased from Hunan Slake Jingda Experimental Animal Co., Ltd. Rats are housed in plastic cages in rooms maintained at 50%–60% relative humidity and 20−23°C, with a 12-hr cycle of light and dark, and all rats are allowed to eat and drink freely. Animal experiments have been approved by the Animal Ethics Committee of Guilin Medical College (GLMC-IACUC-2022032) and comply with the 8^th^ edition of the Guidelines for the Care and Use of Experimental Animals. In this experiment, 36 rats were randomly divided into six groups as follows: control group, EsA group, AP group, AP + EsA group, AP + PDTC group, and AP + EsA + PDTC group, 3–6 rats per group.

### 2.2. Construction of Acute Pancreatitis Model

The establishment of the AP model was carried out according to the methods reported in the literature [14]. Rats fasted for 18 hr before inducing pancreatitis, and were intraperitoneally injected with cerulein (50 *µ*g/kg, MCE, HY-A0190, USA) every 1 hr, a total of six times. After the sixth injection, LPS (10 mg/kg, Solarbio, Beijing, China) was injected intraperitoneally. Control group: without any treatment. EsA group received 10 mg/kg EsA once a day for 7 days. The AP group was given an equal volume of physiological saline by gavage 24 hr after the end of modeling, once a day, for seven consecutive days. AP + EsA group was given 10 mg/kg EsA by gavage 24 hr after the end of modeling, once a day, for seven consecutive days. The AP + PDTC group was given 15 mg/kg PDTC by gavage 24 hr after the end of modeling, once a day, for seven consecutive days. In the AP + EsA + PDTC group, rats were given 10 EsA and 15 mg/kg PDTC by gavage 24 hr after the end of modeling, once a day, and sacrificed over seven consecutive days. After that, the rats were sacrificed.

### 2.3. Cell Culture

RAW264.7 cells were grown in DMEM containing 10% fetal bovine serum (FBS; Sigma, USA), 100 U/mL penicillin, and 100 g/mL streptomycin (Solarbio, Beijing, China), and cultured in an incubator at 37°C and 5% CO_2_. When the cells grew to 80% confluence, they were seeded at 1 × 105 cells/ml. The seeded plates were then divided into blank control group, LPS group, LPS + EsA group, and EsA group. RAW264.7 cells were purchased from the Cell Bank of the Chinese Academy of Sciences (Shanghai, China). None of the drugs and reagents used were endotoxin free.

### 2.4. Cell Viability Assay

Cell viability was evaluated using a conventional 3-(4,5-dimethylthiazol-2-yl)-2,5-diphenyltetrazolium bromide (MTT) reduction test. Cells were inoculated in a 96-well plate at an appropriate density for 18 hr. Treated with 5, 10, 20, 40, 60 *μ*M EsA, and/or 1 *μ*g/ml LPS for 24 hr, respectively, and then adding 20 *μ*l MTT (5 mg/ml) solution and culturing the cells continuously for 4 hr. Afterwards, the supernatant was carefully removed and 150 *μ*l dimethyl sulfoxide (DMSO) was added to each well for dissolution and the absorption value was read at 490 nm in a microplate reader.

### 2.5. Cell Apoptosis Detection

Detection of apoptosis in inflammatory cells by Annexin V-FITC and PI double-staining assay. The assay was performed according to the method outlined in the kit instructions (BD Pharmingen™ FITC Annexin V Apoptosis Detection Kit I). Inflammatory cell suspensions were collected separately and cells were resuspended at a concentration of 1 × 10^6^ cells/ml in 10x binding buffer. Staining according to the kit instructions, incubate for 15 min protected from light and assay on the machine within 1 hr.

### 2.6. Western Blotting

Cells and tissues from each group were collected and total protein was extracted from each group of cells and tissues. The protein concentration of each group was determined by the BCA method. After electrophoresis, membrane transfer, and blocking with an equal amount of protein, an antibody against phosphorylated-p65 (p-p65; Cell Signaling Technology, 3033, 1 : 1,000; Proteintech, bs-0982R 1 : 1,000), Bax (Abcam, ab32503, 1 : 1,000), and Bcl-2 (Abcam, ab182858,1 : 2,000; Proteintech, 26593-1-AP, 1 : 2,000) was added for incubation overnight. The next day, the membrane was incubated with the horseradish peroxidase (HRP) labeled secondary antibody at room temperature for 1 hr. Protein bands were detected using an ECL luminescent kit. ImageJ software is used for grayscale value analysis. Each sample was provided with three replicate holes.

### 2.7. Enzyme Linked Immunosorbent Assay

After the above treatment, the supernatant was collected and the content of inflammatory factors was measured by enzyme linked immunosorbent assay (ELISA, Elabscience®, Wuhan, China). Blood was collected through the abdominal aorta under sterile conditions, and centrifuged (3,000 *r*) for 10 min to isolate the serum. Fully cut the colon tissue, grind it, add nine times the amount of 0.9% sodium chloride solution, and fully mix it. Collect the mixture in a sterile tube. Centrifuge (3,000 *r*) for 10 min to take the supernatant. The concentrations of TNF-*α* and IL-6 were determined by ELISA using an enzyme marker. The method of determination was strictly in accordance with the kit instructions.

### 2.8. Hematoxylin and Eosin (H&E) Staining and Histological Score

After the rats were killed, pancreatic tissue and a long colon tissue (5 and 2–3 cm from the ileocecal region, respectively) were immediately removed and fixed with 10% formalin for 24 hr. After embedding, dehydration, sectioning, and hematoxylin and eosin (H&E) staining. They were observed under an optical microscope. The pathological score of pancreatic tissue is based on the scoring criteria of Schmidt et al. [15]. The pathological score of intestinal inflammation is based on the degree of intestinal mucosal crypt injury and inflammatory cell infiltration score [16].

### 2.9. Statistical Analysis

All data were processed using the software GraphPad Prism 8.0 and IBM SPSS Statistics 25 and data were assessed using the mean ± standard deviation. An independent sample *t*-test analysis was used between the two groups, and a one-way analysis of variance (ANOVA) was used for comparison between multiple groups to evaluate statistical significance. A *P* value  < 0.05 was considered statistically significant.

## 3. Results

### 3.1. Effects of EsA and LPS on RAW264.7 Cell Viability

**Figure 1:**
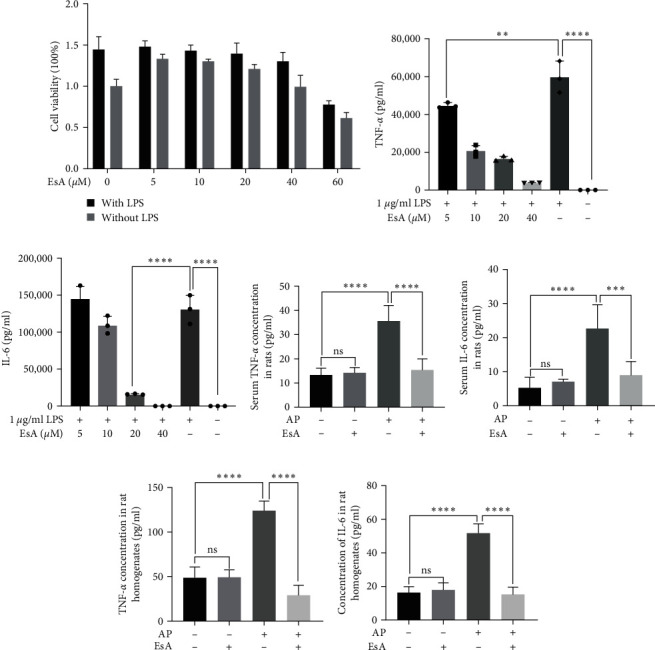
Anti-inflammatory effects of EsA: (a) the cytotoxicity of EsA and/or LPS on RAW264.7 cells by MTT assay, (b, c) the effects of EsA pretreatment on the production of TNF-*α* and IL-6 in LPS-induced RAW264.7 cells, and (d–g) the effects of EsA treatment on the production of TNF-*α* and IL-6 in rat AP serum and intestinal mucosal homogenates. Values are expressed as the means ± SD, *n* = 3 in each group.  ^*∗∗*^*p* < 0.01,  ^*∗∗∗*^*p* < 0.001,  ^*∗∗∗∗*^*p* < 0.0001. EsA, esculentoside A and LPS, lipopolysaccharide.

According to the experimental results when the concentration of EsA reaches 60 *μ*M whether using EsA alone or in combination with LPS, the viability of the cells is lower than 90%. As a result, we subsequently conducted experiments using 0, 5, 10, 20, and 40 *μ*M of EsA and 1 *μ*g/ml of LPS. The results are shown in Figure 1(a).

### 3.2. EsA Can Reduce the Inflammatory Factor TNF-*α* and IL-6 Secretion

TNF-*α* and IL-6 are common inflammatory factors that play an important role in various inflammatory diseases. In this study, the concentrations of TNF-*α* and IL-6 were measured by ELISA to investigate the anti-inflammatory effects of EsA. In the in vitro experiments, we found that EsA inhibits TNF-*α* and IL-6 secretion in a dose-dependent manner (Figures 1(b) and 1(c)). In addition, we obtained the same results in the in vivo experiments, regardless of whether it was rat serum or colon tissue homogenate, TNF-*α* and IL-6 in the AP + EsA group were both lower than those in the AP model group and the difference was statistically significant (*p* < 0.001), while the difference between the AP + EsA group and the control group was not statistically significant, Moreover, there was no significant difference between the EsA group and the control group (Figure 1(d)−1(g)).

### 3.3. EsA Treatment Can Reduce Pancreatic and Intestinal Inflammatory Reactions in Rat AP Model

**Figure 2:**
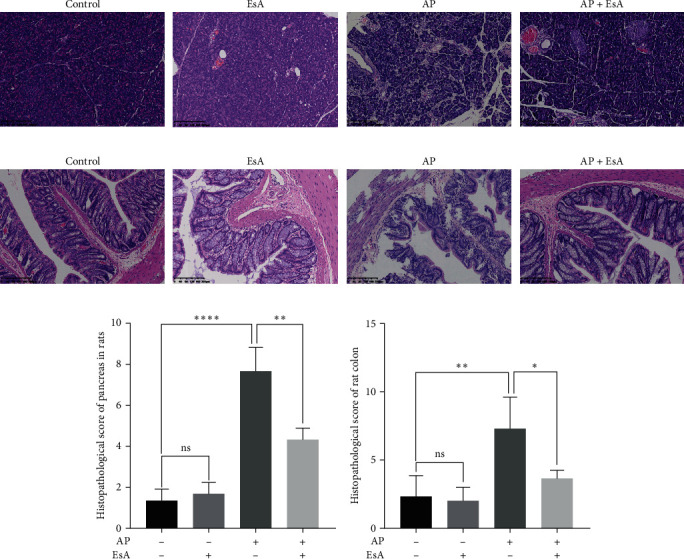
Effects of EsA treatment on inflammation of the pancreas and intestines in SD rats. The rats were induced with cerulein and LPS to establish the rat model of AP: (a, b) the inflammation of pancreas and intestine was investigated by H&E staining and (c, d) histological score. Values are expressed as the means ± SD, *n* = 3 in each group.  ^*∗*^*p* < 0.05,  ^*∗∗*^*p* < 0.01,  ^*∗∗∗∗*^*p* < 0.0001. EsA, esculentoside A; LPS, lipopolysaccharide; AP, acute pancreatitis; and H&E, Hematoxylin and eosin.

The histopathological section showed that the pancreatic acini in the control group were arranged neatly, the lobular parenchymal structure was clear and complete. The structure of glandular lobules and follicles in EsA group was clear and intact, and there was no significant difference compared with the blank control group. In the AP group, large areas of acinar necrosis, disordered arrangement of acinar cells and obvious vacuolization, as well as significant interstitial hemorrhage, and scattered inflammatory cell infiltration between septal connective tissue can be seen. In the AP + EsA group, although the acinar arrangement was slightly poor, there was no significant vacuolar necrosis of acinar cells in the pancreatic tissue. And bleeding in the lobular parenchyma were lighter than those in the AP group (Figure 2(a)). According to the pancreatic pathology scores of each group, it can be seen that the AP group has the highest score, and the AP group has statistical significance compared to the AP + EsA group (*p* < 0.01). The AP group also has statistical significance compared to the control group (*p* < 0.001; Figure 2(c)). In addition, we also evaluated the pathological sections of rat colon tissue, and the results were consistent with pancreatic tissue. The pathological sections of the colon of the control group rats showed clear and complete anatomical structures in each layer. There was no significant difference between the EsA and control groups. The AP group showed incomplete mucosal epithelium, and varying degrees of rupture and defect. The number of intestinal glands decreased and was arranged sparsely. Compared with the AP group, the AP + EsA group showed relatively complete anatomical structure, clear and complete crypt structure (Figure 2(b)). The pathological scoring results showed that the AP group had statistically significant scores compared to the NS group (*p* < 0.05). The comparison score between the AP group and the AP + EsA group also showed statistical significance (*p* < 0.05; Figure 2(d)). The histopathological results indicate that the intestine is affected during AP. In our experiment, it was found that after receiving EsA treatment, both pancreatic inflammation and AP-induced intestinal inflammation were alleviated to a certain extent.

### 3.4. EsA Can Promote Apoptosis of Inflammatory Cells to Alleviate Inflammatory Reactions

**Figure 3:**
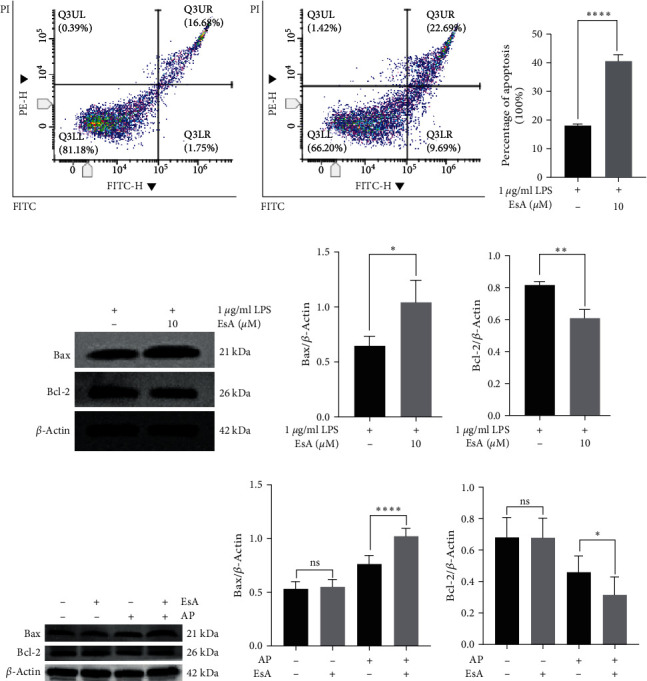
Proapoptotic effect of EsA: (a–c) flow cytometry to detect the effect of EsA treatment of LPS-induced RAW264.7 inflammatory cells on apoptosis, (d–f) the expression of Bax and Bcl-2 in LPS-induced inflammatory cells treated by EsA was detected by WB assay, and (g–i) the expression of Bax and Bcl-2 in intestinal tissues of rats with acute pancreatitis treated by EsA was detected by WB assay. Values are expressed as the means ± SD, *n* = 6 in each group.  ^*∗*^*p* < 0.05,  ^*∗∗*^*p* < 0.01,  ^*∗∗∗∗*^*p* < 0.0001. EsA, esculentoside A and LPS, lipopolysaccharide.

In the in vitro experiments, we used the Annexin V-FITC and PI double staining methods to detect the apoptosis of inflammatory cells. The results showed that compared with the LPS group, the number of apoptotic cells after adding EsA was significantly increased. The results are shown in the figure (Figures 3(a) and 3(b)), and the comparison between the two groups of data was statistically significant (*p* < 0.0001; Figure 3(c)). In addition, in order to further study the mechanism of EsA promoting apoptosis, we analyzed the expression levels of the proapoptotic molecule Bax and the antiapoptotic molecule Bcl-2 in two cell groups, LPS and EsA, using western blotting (WB) experiments, and found that the expression of Bax in the EsA group was significantly higher than that in the LPS group, while the expression of Bcl-2 was opposite (Figure 3(d)−3(f)). Coincidentally, in the in vivo experiments, we found that the expression of Bax in the colon mucosa and submucosa of rats treated with EsA was significantly higher than that of the AP model group, while the expression of Bcl-2 was the opposite. However, there was no difference in the expression of both Bax and Bcl-2 in EsA and control groups (Figure 3(g)–3(i)).

### 3.5. EsA Treatment Reduced Expression of NF-*κ*B Phosphorylated p65

**Figure 4:**
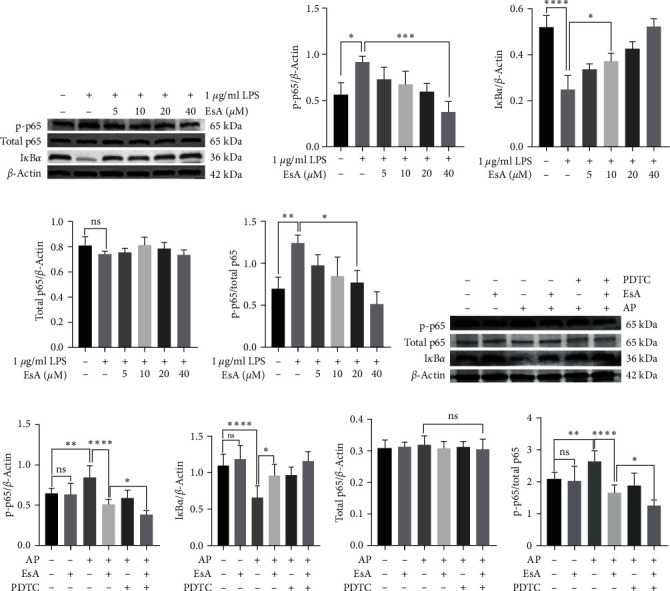
The protein's expression of p-p65, I*κ*B*α*, and total p65 were determined by western blotting: (a–e) the effects of different concentrations of EsA pretreatment on the proteins expression of p-p65, I*κ*B*α*, and total p65 of LPS-induced RAW264.7 cells and (f–j) the proteins expression of p-p65, I*κ*B*α*, and total p65 in intestinal tissues of different animal subgroups. Values are expressed as the means ± SD, *n* = 6 in each group.  ^*∗*^*p* < 0.05,  ^*∗∗*^*p* < 0.01,  ^*∗∗∗*^*p* < 0.001,  ^*∗∗∗∗*^*p* < 0.0001. EsA, esculentoside A and LPS, lipopolysaccharide.

Under normal conditions NF-*κ*B binds to the repressor protein I*κ*B in the cytoplasm as a dimer (mainly p65 and p50 aggregates), when NF-*κ*B is activated I*κ*B is degraded and p-p65 increases into the nucleus. In the in vitro experiments, as shown by the graphs (Figure 4(a)−4(e)), the expression of NF-*κ*B p-p65 decreased with the increase of EsA concentration. Whereas, I*κ*B*α* expression increased with EsA concentration and total p65 expression was unchanged. Furthermore, the same trend was obtained in the in vivo experiments, where the expression of p-p65 in the AP + EsA group was significantly lower than that in the AP model group, and the p-p65 expression in the AP + PDTC group was also reduced but not different from that in the AP + EsA group. Meanwhile, the p-p65 expression in the AP + EsA + PDTC group was lower than that in the AP + EsA + PDTC groups and the same results were seen for I*κ*B*α*, whereas total p65 expression was unchanged (Figure 4(f)−4(j)).

## 4. Discussion

Inflammation is an important defense response in the body, but long-term inflammation can also damage the body. Therefore, it is necessary to suppress the inflammatory response in the body [17]. Stimulating macrophages with LPS can induce classic activation of macrophages, producing inflammatory factors such as TNF-*α*, IL-6, iNOS, COX-2, etc. [18]. So, in this experiment, LPS was used to stimulate RAW264.7 macrophages to construct an inflammatory cell model to verify the anti-inflammatory effect of EsA. TNF-*α*, it is a proinflammatory cytokine that is initially associated with the killing of tumor cells and plays a key role in regulating proinflammatory and anti-inflammatory mediators [19]. IL-6 has both proinflammatory and anti-inflammatory effects. Low concentrations of IL-6 have been detected in healthy individuals, which play a certain role in the body's defense. However, when the concentration of IL-6 in the body increases, it can lead to a series of inflammatory reactions [20]. Our study found that after pretreatment of RAW264.7 cells with EsA, the concentration of TNF-*α* and IL-6 showed a dose-dependent decrease, which is consistent with the research results of Li et al. [21] indicating that EsA has anti-inflammatory effects.

In addition, we also investigated the proapoptotic effect of EsA. Studies have found that EsA can induce apoptosis in mouse glomerular and tubular cells, thereby achieving therapeutic effects on lupus nephritis (LN) [13]. EsA can induce apoptosis in concanavalin A (ConA) activated thymocytes and regulate multiple apoptotic signaling pathways in activated T cells [22]. The key step in cell apoptosis is mitochondrial outer membrane permeability (MOMP), which irreversibly causes cell death. Bax, as a proapoptotic protein, plays a core role by forming mitochondrial outer membrane oligopores, thereby performing MOMP and causing cell apoptosis [23]. According to reports, the antiapoptotic protein Bcl-2 may directly interact with its BH3 domain during the Bax oligomerization process, preventing its homologous oligomerization and inhibiting cell apoptosis [24]. In the experiment, we induced RAW264.7 cells with LPS to establish an inflammatory cell model and treated them with EsA. The results of flow cytometry showed that apoptosis was significantly higher in the EsA group than in the LPS group. Furthermore, WB results showed that the expression of Bax in the EsA group was significantly higher than that in the LPS group, while the expression of Bcl-2 was exactly opposite to Bax, indicating that the anti-inflammatory effect of EsA may be achieved through promoting apoptosis of inflammatory cells.

Shangluo is one of the traditional Chinese medicines in China, which has been shown to possess anti-inflammatory, antibacterial, antiviral, diuretic, proapoptotic and antitumor effects. EsA is a triple saponin compound extracted from the tuberous roots of Shangluo. It has significant anti-inflammatory effects and has been shown to have good anti-inflammatory effects in a variety of acute and chronic inflammatory models. Both Ci et al. [25] and Zhong et al. [12]. demonstrated that EsA had a significant inhibitory effect on airway inflammation in mice. The anti-inflammatory activity of EsA is relatively well characterized, and it has been demonstrated [26] that EsA can result in the blockage of compound-induced production of inflammatory cytokines, thereby sparing tissues from damage, in which different signaling pathways are involved with specific up-regulated and downregulated key components, such as the NF-*κ*B and Nrf2 signaling pathways.

Currently, the occurrence of AP intestinal inflammation is believed to be associated with disturbances in blood circulation, inflammatory factors, and the dysregulation of gastrointestinal hormones. However, the specific mechanisms remain incompletely elucidated, and the inflammatory response plays a crucial role in the development and prognosis of intestinal damage in AP [27]. Liu et al. [28] demonstrated that ligation of the mesenteric lymphatic vessels effectively prevents the entry of intestinal inflammatory mediators into the systemic circulation, thereby restricting their accumulation within the intestinal tract. Consequently, during severe acute pancreatitis (SAP), there is an induction of excessive release of inflammatory mediators and an exaggerated immune response, exacerbating intestinal damage. Research has identified a substantial presence of NF-*κ*B p65-positive cells in the nuclei of intestinal mucosal cells in the SAP rat model, suggesting an early and significant activation of NF-*κ*B in the intestinal mucosa during the initial stages of SAP. Therefore, this study aims to investigate the role of EsA in AP intestinal inflammation.

NF-*κ*B is a nuclear protein factor that binds specifically to the enhancer B sequence of the immunoglobulin *κ* light chain gene [29] and is widely present in human tissue cells, participating in a variety of biological processes including inflammatory responses, immune responses, apoptosis, and tumorigenesis. Under normal circumstances, NF-*κ*B binds to the inhibitory protein I*κ*B in the cytoplasm as a dimer (mainly p65 and p50 aggregates). When endotoxin, oxidation products, lysophosphatidylcholine, and metabolite products activate the I*κ*B kinase (I*κκ*) complex, I*κ*B*α* is phosphorylated, NF-*κ*B dimers dissociate from I*κ*B*α*, and p65 is phosphorylated and translocated to the nucleus to initiate transcriptional expression and promote the release of inflammatory mediators. This study found that EsA can reduce the expression of phosphorylated NF-*κ*B p65 and elevate the expression of I*κ*B*α* in a dose-dependent manner, and there was no change in the total p65 expression, suggesting that EsA may inhibits inflammatory response by inhibit the activation of the NF-*κ*B signaling pathway.

Other than that, we also constructed a rat AP model. In our experiments, we found that the AP + EsA group had lower TNF-*α* and IL-6 than the AP model group in both the peripheral blood supernatant and the homogenate of colonic mucosa and submucosal tissues, which did not differ from the control group, and that the EsA group did not differ from the control group, indicating that EsA can suppress the inflammatory response by inhibiting the secretion of inflammatory factors, which again validates the anti-inflammatory effect of EsA. Our experiment demonstrated the success of AP model modeling by using H&E staining. The pathological results showed significant inflammatory reactions in both pancreatic and colon tissues in the AP group, indicating successful modeling. The inflammatory reaction of the colon is mainly distributed in the mucosa and submucosa of the colon. In addition, WB results showed that the expression of Bax in the colonic mucosa and submucosa tissues of rats in the AP + EsA group was higher than that in the AP group, while the expression of Bcl-2 showed the opposite result, which was consistent with the results of cell experiments, indicating that EsA might inhibit the inflammatory response by promoting apoptosis of inflammatory cells. Moreover, there was no difference in the expression of either Bax or Bcl-2 between the EsA group and the control group, and its proapoptotic outcome may only play a role in the disease state. Additionally, in order to further verify the anti-inflammatory mechanism of EsA, we have not only set up the control group, EsA group, AP group, AP + EsA group, but also set up the AP + PDTC group and AP + EsA + PDTC group. PDTC inhibits I*κ*Ba degradation and is widely used as a specific inhibitor in the study of the NF-*κ*B pathway. Our WB experiment results showed that the expression of p-p65 was significantly inhibited in the AP + EsA + PDTC group, whereas the expression of I*κ*B*α* was significantly elevated, while the total p65 expression was unchanged. It is well known that when the NF-*κ*B signaling pathway is activated p65 is phosphorylated and the p-p65 enters the nucleus to initiate transcriptional expression, so lowering the expression of p-p65 and elevating the expression of I*κ*B*α* can inhibit the activation of the NF-*κ*B signaling pathway and thus play an anti-inflammatory role.

However, there was no further evidence in this experiment about whether the proapoptotic mechanism of EsA is associated with the NF-*κ*B pathway, and future experiments could further explore whether there exists is some correlation between the proapoptotic mechanism of EsA and the NF-*κ*B pathway.

## 5. Conclusions

In summary, our study shows that EsA inhibits the secretion of inflammatory factors TNF-*α* and IL-6 by regulating the NF-*κ*B signaling pathway and proinflammatory cell apoptosis in LPS-induced inflammatory cells and rat models of AP, respectively, providing evidence that EsA may be a novel therapeutic agent for the treatment of AP-induced intestinal inflammation and further arresting AP progression. However, the pathogenesis of AP is extremely complex, involving multiple inflammation-related factors and implicating multiple organs. Moreover, the manner in which EsA regulates the NF-*κ*B signaling pathway is unclear. More animal and clinical trials are needed in the future to further explore the efficacy and safety of EsA in the treatment of intestinal inflammation in AP.

## Data Availability

The data used to support the findings of this study are included within the article.

## Abbreviations

AP: Acute pancreatitis
SIRS: Systemic inflammatory response syndrome
MODS: Multiple organ dysfunction syndrome
EsA: Esculentoside A
LPS: Lipopolysaccharide
PDTC: Plymouth and Devonport Technical College
FBS: Fetal bovine serum
MTT: 3-(4,5-dimethylthiazol-2-yl)-2,5-diphenyltetrazolium bromide
DMSO: Dimethyl sulfoxide
WB: Western blotting
p-p65: phosphorylated-p65
HRP: Horseradish peroxidase
ELISA: Enzyme linked immunosorbent assay
H&E: Hematoxylin and eosin
LN: Lupus nephritis
MOMP: Mitochondrial outer membrane permeability
PLA2: Phospholipase A2
PAF: Platelet-activating factor
LT: Leukotriene
PGE: Prostaglandin E
TXA2: Thromboxane A2.
